# Sequential Immunization With Heterologous Viruses Does Not Result in Attrition of the B Cell Memory in Rainbow Trout

**DOI:** 10.3389/fimmu.2019.02687

**Published:** 2019-11-19

**Authors:** Sofie Navelsaker, Susana Magadan, Luc Jouneau, Edwige Quillet, Niels J. Olesen, Hetron Mweemba Munang'andu, Pierre Boudinot, Øystein Evensen

**Affiliations:** ^1^Department of Basic Sciences and Aquatic Medicine, Faculty of Veterinary Medicine, Oslo, Norway; ^2^VIM, INRA Centre Jouy-en-Josas, Jouy-en-Josas, France; ^3^Centro de Investigaciones Biomédicas (CINBIO), University of Vigo, Vigo, Spain; ^4^GABI, INRA, AgroParisTech, Université Paris-Saclay, Jouy-en-Josas, France; ^5^DTU Veterinary Institute, Technical University of Denmark, Kongens Lyngby, Denmark

**Keywords:** antibodies, B cell repertoire, heterologous immunization, public response, fish immunology, RepSeq, comparative immunology

## Abstract

Long-term immunity is of great importance for protection against pathogens and has been extensively studied in mammals. Successive heterologous infections can affect the maintenance of immune memory, inducing attrition of T memory cells and diminishing B cell mediated protection. In fish, the basis of immune memory and the mechanisms of immunization to heterologous pathogens remain poorly understood. We sequentially immunized isogenic rainbow trout with two immunologically distinct viruses, VHSV and IPNV, either with one virus only or in combination, and analyzed the antibody responses and repertoires. Neutralizing antibodies and ELISPOT did not reveal an effect of heterologous immunization. Using a consensus read sequencing approach that incorporates unique barcodes to each cDNA molecule, we focused on the diversity expressed by selected responding VH/C combinations. We identified both public and private responses against VHSV and/or IPNV in all groups of fish. In fish immunized with two viruses, we registered no significant reduction in the persistence of the response toward the primary immunization. Similarly, the response to the second immunization was not affected by a prior vaccination to the other virus. Our data suggest that heterologous immunization does not enforce attrition of pre-existing antibody producing cells, which may impair the protection afforded by multiple successive vaccinations. These observations are potentially important to improve vaccination strategies practiced in aquaculture.

## Introduction

Adaptive immune response to infections leads to the selection of memory B and T cell populations, resulting in cell subsets of limited size controlled by homeostatic mechanisms (1, 2). Multiple infections over time produce new memory cells entering the general pool, leading to a successive partial replacement of pre-existing memory cells. Mechanisms controlling the maintenance and homeostasis of cell populations responsible for immunological memory are poorly understood. Sequential responses to heterologous pathogens is therefore of particular interest since different infections and responses can lead to enhanced or diminished (attrition) protective immunity (3).

Memory cell attrition was first studied for T cells. In a pivotal report published in 2004 (4), Selin et al. showed selective loss of LCMV-specific memory CD8^+^ T cells after sequential infections with heterologous viruses (5). Altered CD4^+^ responses have also been reported, for example in nurses frequently exposed to viral pathogens (6). Two models have been proposed to explain T cell attrition. First, passive competition and replacement of pre-existing cells by new memory cells entering a compartment of limited size from the memory compartment in concert with reactivation of cross-reactive memory cells (4). Alternatively, active deletion of pre-existing memory cells through apoptosis (7). Underlying mechanisms are not understood, but type I IFN has been implicated to play a role. It was shown that apoptosis and memory loss did not occur in mice with disrupted IFN receptor, and type I IFN thus appeared to be required for the depletion of pre-existing anti-LCMV memory T cells (8, 9). It has also been proposed that the memory CD8^+^ T cell compartment becomes larger over time, resulting in a progressive increase of the capacity to include larger numbers of clonotypes (hence, specificities) (10). TCRs are highly cross-reactive, and memory T cells often cross-react with newly encountered pathogens. Hence, it could happen that cross-reactive T cells dominate a given T cell response, thus hampering the selection of potentially more protective clones (9). The attrition of T memory cells induced by type I IFN at the onset of new viral infections could therefore facilitate highly diverse T cell responses to each new viral pathogen encountered by the host and reduce the effect of heterologous immunity (3).

Antibodies (Abs) are usually more specific for their epitope than TCR. The memory B cell pool and the antibody-dependent protection may therefore evolve quite differently after heterologous infections compared to T cells. Antibody-based immunity is very long-lasting against several pathogens. No decay of antibody-based immunity against measles and mumps was observed, suggesting a life-long protection after recovery from primary infections from these viruses (11). Also, the contrasted fractions of adults, previously exposed to different influenza viruses, and children, developing influenza during the 1957 pandemic, suggests that accumulated heterosubtypic immunity largely influences the antibody dependent protection (12). However, the longevity of antibody memory and the magnitude of associated anamnestic responses is heterogeneous across individuals (13), and pathogen dependent. Besides the effect of aging, and the passive and progressive reduction of the memory cell pool by replacement during responses to infections, additional mechanisms for memory B cell attrition have been identified recently. Loss of pre-existing memory B cells after parasitic infections have been reported in several models. Radwanska et al. found that trypanosomes not only delete several B cell populations and inhibits the establishment of a long-term specific protection by Abs, but also abrogate host DTPa vaccine induced memory response (14). Pre-existing immunity against Influenza A virus was also abrogated by a single malaria infection, with loss of long-lived plasma cells and virus specific Abs (15). However, this loss was not definitive and both Influenza virus specific long-lived plasma cells and Ab titers were restored with time, suggesting that memory B cells may play a role in recovery from attrition induced by heterologous infection (15). More recently, another mechanism for deletion of B cells directed against the glycoprotein of LCMV, a model for chronic viral infection in mice, was demonstrated (16). After adoptive transfer of LCMV specific B cells, mice mounted a fast response after infection which rapidly declined. The loss of activated specific B cells was mediated by type I IFN response induced soon after infection but did not require IFN signaling in B cells. This B cell decimation was apparently due to a pathway, favoring differentiation of antigen-specific B cells into short-lived antibody secreting cells (ASC). Altogether, these observations illustrate the diversity of mechanisms involved in B cell attrition in the context of heterologous infections in mammals.

Attrition of B cell response after successive heterologous infections, or immunizations, has not been documented in fish. Such mechanisms are most likely affected by the particular characteristics of fish B cell responses. The respective importance of T-dependent and T-independent B cell responses against pathogens is certainly crucial, but it is poorly documented. In absence of lymph nodes and typical germinal centers, the microenvironments of B cell differentiation and activation also remain ill-defined in fish. Different types of Ab producing cells have been identified: plasma blasts secrete low amounts of Ab, are able to divide, and express low levels of membrane Ig, while long- or short-lived plasma cells secrete high amount of Ab, do not replicate and do not express Ig at the cell surface (17, 18). The definition of a fish memory B cells remains elusive in absence of specific markers (19). After B cells differentiate into ASC upon encounter with their specific antigen (Ag), they mature into plasma cells that accumulate and persist in the anterior kidney (20). In absence of typical germinal centers, affinity maturation of fish Ab response is of limited amplitude, as reported in frogs (21, 22). However, strong specific B cell responses have been observed against many pathogens in multiple fish species, and neutralizing Abs are critical for protection against viruses. Efficient vaccines have been designed to protect fish against numerous bacterial and viral diseases by inducing high titers of protective specific serum Abs (23–25). Vaccination of farmed fish has expanded since the early eighties, mainly against bacterial diseases, and development of commercial vaccines against viral diseases is expected to grow. In this context, understanding the effect of sequential immunizations on memory compartments and long-term protection, as well as the consequences of heterologous immunizations in fish, is important.

We have previously characterized the persistent Ab response induced by an attenuated vaccine against the rhabdovirus VHSV in rainbow trout (*Oncorhynchus mykiss*). Vaccination led to high serum titres of neutralizing Abs which persisted for at least 1 year. When modifications of the spleen Ig repertoire were analyzed, private responses (i.e., found in a minority of individuals) as well as public responses [i.e., found in (almost) all individuals] were identified and persisted for months (26). These observations, especially the public response, provide a useful system to test the effect of subsequent immunizations on pre-existing Ag-specific Ab producing cells *at the clonotype level*.

In this paper, we asked whether a secondary immunization with a heterologous virus, administered after a primary vaccination with a live VHSV vaccine, modifies the B cell mediated response to the primary antigen (VHSV). We chose a model where trout vaccinated with live VHSV were subsequently immunized with an immunologically distinct birnavirus (IPNV) vaccine. Our results indicate that secondary IPNV immunization does not lead to a drastic modification of the B cell response elicited by the primary vaccination against VHSV.

## Materials and Methods

### Fish Vaccination and Ethical Statement

**Figure 1:**
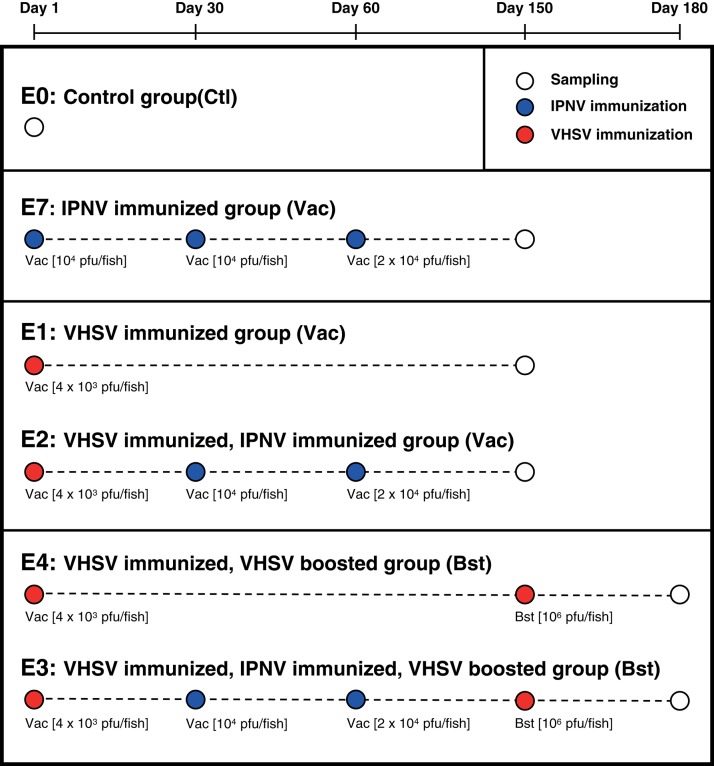
Experimental plan. Six groups of fish were immunized following different protocols. Sampling was performed at different times indicated with white nodes. The red nodes indicate vaccination with VHSV, blue nodes indicating vaccination with IPNV, and sampling is indicated with white nodes. Viral loads were between 4000 and 10^6^ pfu/fish administrated i.m. (VHSV) or i.p. (IPNV).

Experimental groups of rainbow trout [isogenic line B57 (27), about 150 g] were kept in separate tanks at 16°C in the fish facilities of Institut National de la Recherche Agronomique (INRA, Jouy en Josas, France). The experimental design is shown in Figure 1. Group 1 (E1) was immunized with VHSV once and sampled 5 months later. Group 4 (E4) was immunized with VHSV, boosted after 5 months, and sampled 1 month later. Group E2 was immunized with VHSV (prime) followed by 2x IPNV immunizations at 30 and 60 days post prime, then sampled 5 months after the first immunization. Group E3 was subject to the same initial immunization protocol, then boosted with VHSV 5 months after the first immunization and sampled 1 month later. Finally, group E7 was immunized three times with IPNV (1 month apart) and sampled 5 months after the first immunization. The control group (i.e., PBS injected) (E0) was sampled prior to onset of the study. The VHSV used for vaccination (i.m. injection, 10^4^ pfu/fish) and boost (i.m. injection, 10^6^ pfu/fish) was the attenuated 25-111 variant of strain 07-71 (28). The IPNV virus, IPNV-TA (rNVI-015), a clone derived from the Norwegian Sp strain NVI015 (Genbank accession No. AY379740) was used for immunization. It is highly virulent and immunogenic to Atlantic salmon (29, 30). IPNV-TA is named after its VP2-capsid amino acid residues T^217^ and A^221^, a virulence marker in Atlantic salmon, while this strain is not pathogenic to rainbow trout (O.E., own observations). Fish immunized with IPNV-TA received an intraperitoneal injection of 1 × 10^4^ PFU/fish (Figure 1). Trout were sacrificed by overexposure to 2-phenoxyethanol diluted 1/1000. Blood was extracted and let to clot at 4°C overnight for serum extraction. The spleen was removed, fixed in RNA later (SIGMA, Aldrich) and kept at −20°C. Serum extraction was performed by centrifugation at 200 × g for 10 min, supernatants were collected and centrifuged at 1000 × g for 20 min, and frozen at −20°C to use in titration assays.

This study was carried out in accordance with the recommendations of the European Union guidelines for the handling of laboratory animals (http://ec.europa.eu/environment/chemicals/lab_animals/index_en.htm). All animal work at INRA was approved by the Direction of the Veterinary Services of Versailles (authorization 78-28) as well as fish facilities (authorization B78-720), and the experimental protocols were approved by the INRA institutional ethical committee “Comethea” (permit license number #15-60).

### Elispot and Virus Neutralization Assay

VHSV specific IgM secreting cells were determined in head kidney using *ex vivo* ELISPOT (31). Mononuclear from head kidney were isolated by centrifugation on density gradient using Histopaque 1077 (Sigma-Aldrich). After washing, 5 × 10^6^, 2.5 × 10^6^, and 1 × 10^6^ cells were plated on wells containing a VHSV-coated nitrocellulose membrane. Coating was performed overnight at 4°C with 200 μl of purified VHSV (75 μg/mL) in PBS. Remaining sites were then blocked with 5% milk in PBS for 1 h at room temperature. To detect VHSV-specific IgM bound to the virus, membranes were incubated for 1 h in the presence of primary monoclonal antibody anti-trout IgM (clone 1.14, 1 μg/mL). After washing, membranes were incubated for 1 h with secondary Ab goat anti-mouse IgG-Biotin (Amersham), then with Avidin-HRP. Finally, HRP activity was revealed by incubation at 20°C with 3-amino-9-ethyl-carbazole (AEC system), until spots appeared. The reaction was finally stopped before readout.

Virus neutralization assay in the presence of trout complement was performed in 12-well plates as previously described (32). Briefly, VHSV was pre-incubated overnight at 4°C with dilutions of the trout serum to be tested, in presence of trout complement. Each serum-complement-virus mixture was then adsorbed for 1 h at 14°C on a monolayer of EPC cells (0.1 ml per well). Two replicates were performed. Cell cultures were then overlaid with 1% methylcellulose medium and incubated for 72 h at 14°C. Finally, after fixing and staining with 0.5% crystal violet, plates were air-dried and plaques were counted. The neutralizing titer was estimated as the highest serum dilution causing a 50% reduction of the average number of plaques counted in control cultures inoculated with control (non-immune) trout serum, virus and complement. Final serum dilutions tested were 1/1000, 1/4000, and 1/16000.

### Sequencing and Data Analysis

Sequencing consisted in paired-end 2 × 300 pb runs, using a MiSeq instrument (Illumina) and the MiSeq Reagent Kit v3 (600 cycles) (Illumina). Sequencing analysis and annotation, estimation of error rate, and normalization by subsampling, as well as validation of our barcoded IgH cDNA sequencing approach, are described (26).

#### RNA Isolation

Total RNA from individual spleens was obtained by following a standard protocol using 1/1.2 mm ceramic beads (Mineralex, France) and TRIzol (Life Technologies, Les Ulys, France). The disruption protocol to homogenize the tissue was pulses for 20 s at 10 000 rpm in a homogenizer (FastPrep 24 G5, MP Biomedicals, Santa Ana US). Samples were then centrifuged, and the top phase containing the RNA was transferred to columns and further purified and DNase treated using RNA extraction kit (QIagen).

#### Illumina MiSeq Libraries Preparation and Sequencing

Libraries for Illumina deep sequencing were prepared as described (26). For cDNA barcoding, the primers used for second strand cDNA contained 15 random nt (Table S1). The location of the first Cμ primer in the Cμ2 domain restricts amplification of IgHm mRNA, since IgHδ contains a Cμ1 domain. The resulting ds cDNA was amplified by PCR to add the Illumina adaptor sequences and to integrate a fish-specific index or barcode. The following VH/C combinations were analyzed: VH4/Cμ (primer VH4.1), VH5/Cμ (primer VH5.1), VH8/Cμ (primer VH8.1), VH4/Cτ (primerVH4.1), and VH5/Cτ (primer VH5.4). Final PCR with Illumina adapters were purified using Agencourt AMPureXP beads (Beckman Coulter, Brea, CA). Library quality was assessed on a “DNA High Sensitivity” chip with a bioanalyzer instrument (Agilent Technologies, Santa Clara, CA). Equal amounts of libraries were pooled for multiplexing, and pools were sequenced in paired end 2 × 300 pb runs using a MiSeq instrument (Illumina) and the MiSeq Reagent Kit v3 (600 cycles) (Illumina) according to the manufacturer recommendations.

This consensus read sequencing approach based on the incorporation of a unique random barcode (UID) in each cDNA molecule produced at the reverse transcription step permits an accurate quantification of clonotype frequencies, and a better correction of PCR/sequencing errors (26).

#### Sequencing Analysis and Annotation

Consensus sequence from read pairs were computed and the CDR3 sequences were extracted as described in Magadan et al. (26). J segments were annotated according to IMGT (http://www.imgt.org/) gene tables (32); one mismatch was allowed in the J (nucleotide) sequences to name the segment. For each consensus, we therefore produced an annotation based on the random barcode (UID) a VH subgroup defined by the V primer used for the amplification, a C type (μ or τ), an in-frame CDR3 sequence, and a J segment. Our V and C annotation defines V family subsets and isotypes, respectively, rather than unique genes. Since we used an isogenic clone of rainbow trout in which every locus is in homozygous configuration, the complexity of annotation was limited to a single haplotype.

#### Definition of a Unique cDNA Molecular Barcoding to Determine Clonotype Count

Amplification bias is a major issue for producing accurate descriptions of immune repertoires using deep sequencing. To solve this problem, different systems of cDNA barcoding have been developed with random UIDs incorporated on one or both side(s) of the templates during reverse transcription. To address PCR biases, we developed a unique molecular identifier (MID) based on the combination of (1) the 15nt random barcode (UID) incorporated in the primer used for the second strand cDNA synthesis (see above) and (2) the CDR3 sequence. Each clonotype being defined by a V tag, a C tag, a J annotation, and a CDR3 amino acid sequence; its expression level was then measured by counting the corresponding MID barcodes.

#### Identification of “public” Clonotypes

Clonotypes participating in “public” or “highly shared” responses were identified as clonotypes being (1) ***detected*** in at least three fish per group, either in vaccinated or boosted groups (“**shared clonotypes**”) and (2) **overexpressed** more than *k* times in average between control and vaccinated (or boosted) fish. We studied the responses detected with *k* = 50, 25, and 10. In a third step, we examined whether the level of expression of these **overexpressed shared clonotypes** was consistently high across immunized fish of the vaccinated and boosted groups. Clonotypes being present in most individuals of immune groups, differentially expressed between controls and immunized fish, and **well-expressed across groups immunized with a virus**, were considered involved in a public response to this virus.

Sequence data used in this work are registered in the BioProject NCBI database with the SRA accession numbers: SRP128087 and PRJNA530387.

## Results

### Neutralizing Antibodies and ELISPOT Do Not Reveal Drastic Effect of Heterologous Viruses

**Figure 2:**
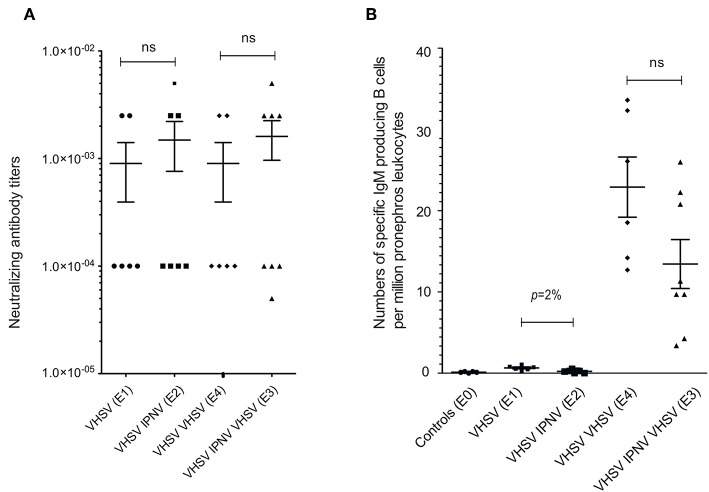
Serum Neutralizing anti-VHSV Abs titres and VHSV ELISPOT. Neutralizing Abs titers in serum assessed by plaque assay **(A)** from groups E1 (sampling 5 months after VHSV vaccination), E2 (sampling 5 months after VHSV vaccination followed by IPNV immunization), E4 (sampling 1 month after VHSV boost of fish vaccinated with VHSV 5 months earlier), and E3 (sampling 1 month after VHSV boost of fish vaccinated with VHSV and later immunized with IPNV). The neutralizing titer was estimated as the highest trout serum dilution causing a 50% reduction of the average number of plaques in control cultures inoculated with control trout serum, virus and complement. See Figure 1 for an overview of experimental design. **(B)** ELISPOT assay detecting head kidney VHSV specific, IgM-producing cells from the E0-E4 groups. Mann–Whitney nonparametric statistical tests were performed (two-tailed). Means and SEM are shown.

First, we analyzed the VHSV neutralizing Abs (nAbs) titers in VHSV-immunized fish. A strong neutralizing activity was detected all immunized fish but titres were not significantly different between fish immunized with VHSV once (E1), twice (E4), or VHSV (prime) followed by 2x IPNV (E2) (Figure 2A). Also, the group given VHSV as prime followed by 2x IPNV and then given a second VHSV boost (E3), (Figure 1) did not show any difference in anti-VHSV nAb titers (Figure 2A). These findings are concordant with previous studies showing that VHSV boost does not modify nAb titers in VHSV primed trout (26). Further, findings reported here indicate that IPNV immunization post VHSV prime does not significantly impact on nAb titers to VHSV (Figure 2A).

The number of VHSV-specific Ab-producing cells in head kidney measured by ELISPOT was low 5 months after primary VHSV immunization, but increased after boost (E1 vs. E4, Figure 2B). We found that IPNV immunization post VHSV prime, reduces the actual number of Ab producing cells, however, not significantly (E3 vs. E4, Figure 2B).

### Fingerprints of IPNV Vaccination in Selected VH/C Repertoires

The prime goal in this study was to find out whether a secondary immunization with IPNV (heterologous) administered post primary VHSV vaccination would modify the response to the primary antigen. Therefore, we first characterized the repertoire post IPNV immunization (group E7) and focused on the VH/C combinations characterized earlier in VHSV immunized fish: VH4/Cμ, VH5/Cμ, VH8/Cμ for IgM and VH4/Cτ and VH5/Cτ for IgT were included.

**Figure 3:**
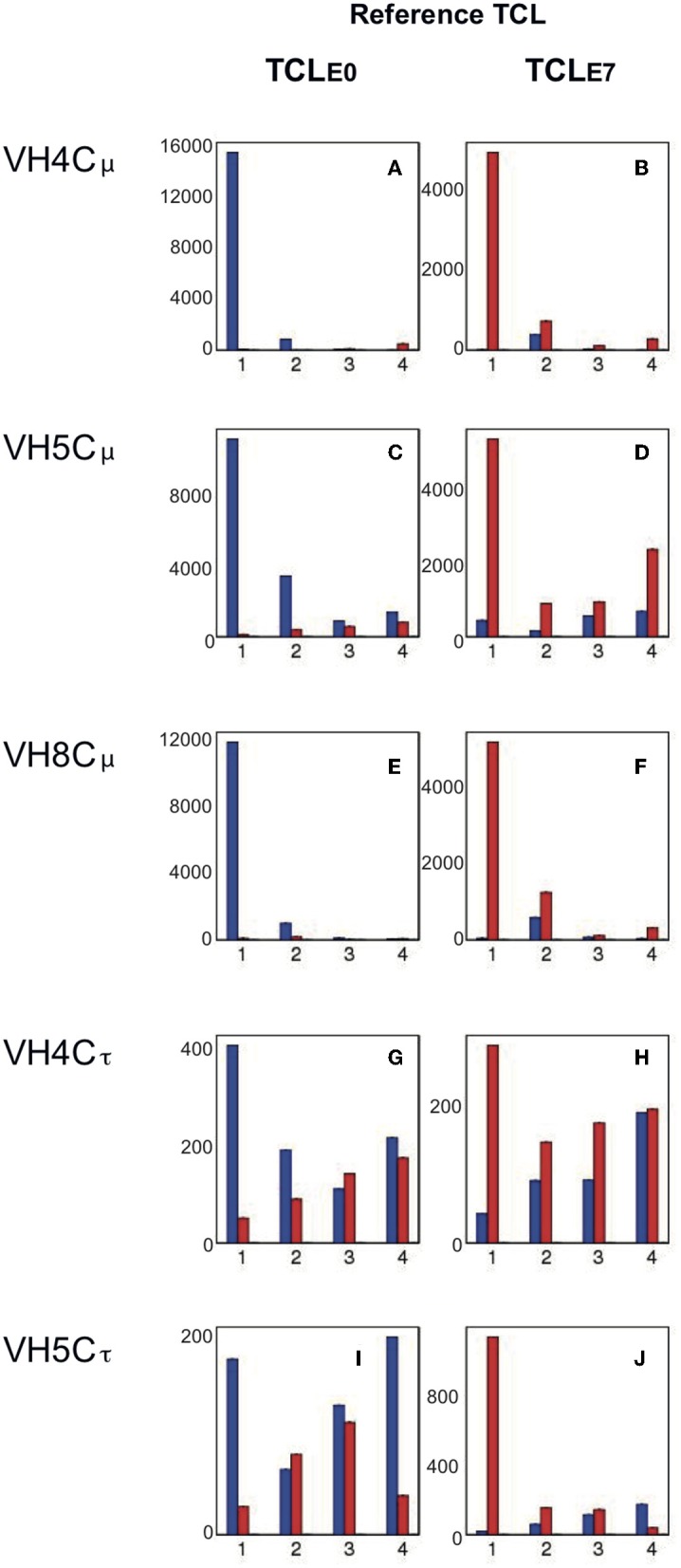
Cumulative expression of Top50 clonotypes shared by n individuals within control (E0) and IPNV (E7) immunized groups. Lists of Top50 clonotypes (TCL) are defined for a group (E0 or E7), as the non-redundant union of the lists of TOP50 clonotypes from the 4 fish belonging to the group. The cumulative expression and sharing of clonotypes from the TCL_E0_ reference list are represented in the left column **(A,C,E,G,I)**, while clonotypes from the TCL_E7_ list are analyzed in the right column **(B,D,F,H,J)**. Bar plots show the total expression and sharing of the 50 most frequent clonotypes of each fish group, either among E0 (in blue) or among E7 (in red): bars represent the cumulative expression of clonotypes found in only one fish or in 2, 3, or 4 fish, respectively. For example, in panel a, blue bars show the cumulative counts of VH4/Cμ clonotypes from TCL_E0_ in control fish, while red bars show cumulative expression of the same TCL_E0_ clonotypes in fish from the IPNV vaccinated group. Similarly, in panel b, red bars show the cumulative counts of VH4/Cμ TCL_E7_ clonotypes in IPNV-immunized fish, while blue bars show cumulative expression of the same TCL_E7_ clonotypes in control fish. Bars are computed from the average values corresponding to top clonotypes found in 1–4 fish, over 10 subsamplings of 7,000).

IgH repertoires in IPNV-immunized fish (E7) were compared to control (E0). Random subsampling of 7000 MIDs per individual fish datasets obtained for each VH/C combination without replacement was done, allowing quantitative comparisons despite variations of the total MID count per group. We focused on the most expressed clonotypes of each fish and for each VH/C (“Top 50” clonotypes), checking their sharing by different individuals within the control group (E0) and in IPNV-immunized fish (group E7) as in Magadan et al. (26). We first defined the list of the Top50 clonotypes (TCL) for each group: for E0 (TCL_E0_) and for E7 (TCL_E7_). To visualize the importance of large shared clonotypes across different fish, we represented as a bar plot the cumulated frequency of these Top50 clonotypes present in all four individuals, or only in 3, or only in 2 or only 1 fish, within a given group (E0 or E7). Thus, Figure 3 depicts the cumulated expression of Top50 clonotypes from E0 (TCL_E0_) present in 1 to 4 individuals belonging to this group (Figures 3A,C,E,G,I; blue bars); the cumulated expression of these TCL_E0_ clonotypes is also represented across fish from the E7 group (Figures 3A,C,E,G,I; red bars). Similarly, we show the cumulated expression of Top50 clonotypes from E7 (TCL_E7_) present in 1-4 fish from this group (Figures 3B,D,F,H,J; red bars), as well as their distribution in fish from E0 (Figures 3B,D,F,H,J; blue bars).

Three VH/C combinations point in the direction of IPNV fingerprints post immunization. These are; (1) for VH4/Cμ (Figures 3A,B) where Top50 clonotypes from control fish are almost all present in only one individual. For IPNV immunized fish abundant clonotypes are found in 2 (sometimes 3 or 4) individuals (see also Figure S1A). While modest, the convergence indicates a clonal response occurring in some, but not all fish as suggested by CDR3 spectratypes (data non shown). (2) for VH5/Cμ, Top50 clonotypes from the control group were expressed at low levels, with some variation between fish (Figure 3C, blue bars). In contrast, a large proportion (about 1/3) of the Top50 clonotypes of the IPNV-immunized fish was shared by 4 IPNV-immunized fish, denoting a public response (Figure 3D, red bars). (3) for VH5/Cτ, the response was not disclosed by convergence but by the expression level of Top50 clonotypes. The cumulated expression of Top50 clonotypes from the control group represented about 450 MID (Figure 3I, blue bars), where a large proportion was shared by >2 fish, as generally found for IgT (26). Opposed to this, Top50 clonotypes from the IPNV group represented a cumulated expression >1400 MID (Figure 3J), for roughly a similar number of clonotypes (see Figure S1). Notably, these clonotypes were each expressed in one fish only, indicating that these responses were essentially private. The different patterns of these responses are also shown in Figure S2 where the expression and sharing of all clonotypes are represented in parallel to the Top50.

We then looked for VH5/Cμ public clonotypes that would be (1) highly shared within the IPNV-immunized group (i.e., present in 3 or 4 individuals), (2) differentially expressed compared to controls (average FC> 10) and (3) detected at least 5 times in > 3 IPNV immunized fish. Two VH5/JH5 sequences were identified that fit these three criteria: CARYSGYNAFDYW (CDR3 = 8AA; Fold Change (E7/E0 = 32) and CARYTGYAFDYW (CDR3 = 7AA; Fold Change (E7/E0 = 12), suggesting that the response to IPNV comprised public components expressing VH5/JH5 rearrangements.

Altogether, these data show that IPNV immunization induces detectable modifications in the IgH repertoire for several of the VH/C combinations.

### IPNV Immunization Post VHSV Vaccination Induces Minor Modifications to the VHSV-Specific Repertoire

Next, we analyzed the modifications to a prime VHSV repertoire induced by secondary IPNV immunization. Repertoires were analyzed 5 months after VHSV vaccination with or without IPNV immunization (Figure 1, E1 and E2). Here we focused on VH5/Cμ and VH4/Cτ combinations. Public VH5/Cμ clonotypes are considered VHSV-specific based on the fact that they are recurrent in VHSV vaccinated fish (26, 33). Further, modifications of the VH5/Cμ repertoire should reflect the responses to both VHSV and IPNV, since VH5/Cμ response were also observed to the latter (Figure 3D). In addition, previous findings showed that modifications of the distribution of clonotype frequency suggested that the VH4/Cτ combination was involved in the VHSV response through large private expansions (33).

**Figure 4:**
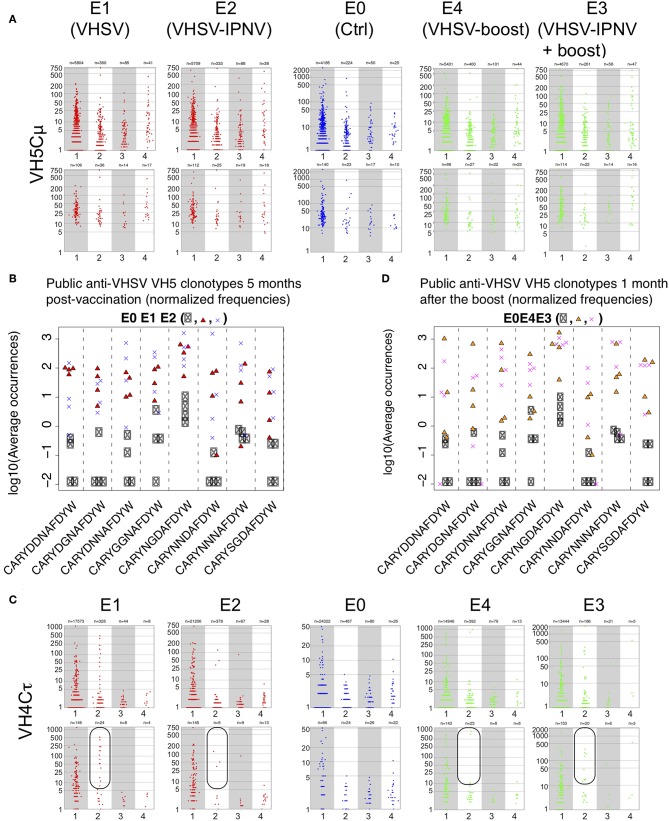
Effect of IPNV immunization on VH5/Cμ and VH4/Cτ repertoires in VHSV vaccinated fish. **(A)** Size distribution of VH5/Cμ clonotypes shared by individual sequenced repertoires from groups E0, E1, E2, E3, and E4. Size distribution is shown for clonotypes found in n fish (*n* = 1, 2, 3, 4), within a subsampling of 7000 MID per fish. For clonotypes found in several fish, the average size is shown. Top panels reflect the results obtained with full repertoire and bottom panels represent TOP50 clonotypes only. **(B)** Normalized frequencies of public VHSV responding VH5/Cμ - clonotypes in E0, E1, and E2 groups (5 months after vaccination). **(C)** Size distribution of VH4/Cτ clonotypes shared by individual sequenced repertoires from groups E0, E1, E2, E3, and E4. Size distribution is shown for clonotypes found in n fish (*n* = 1, 2, 3, 4), within a subsampling of 7000 MID per fish. Top panels reflect the results obtained with full repertoire and bottom panels represent TOP50 clonotypes only. **(D)** Normalized frequencies of public VHSV responding VH5/Cμ - clonotypes in E0, E3, and E4 groups (after the boost).

The size and sharing distribution of VH5/Cμ clonotypes did not differ between E1 (VHSV only) and E2 (VHSV + 2x IPNV immunizations, Figure 4A). This applied to the whole repertoire and the Top50 clonotypes, suggesting that a secondary IPNV immunization did not have a drastic effect on the pre-existing VHSV repertoire (Figure 4A). This observation was also supported by the expression analysis and number of shared clonotypes (Figure S3, A - b vs. h, B - b vs. h). Previously (26), it was shown that VHSV immunization elicits 8 public clonotypes, and for group E2, we questioned whether IPNV immunization changed the relative frequency of these 8 public clonotypes. This turned out not to be the case, and the 8 clonotypes expanded and are shared in all vaccinated fish to the same level (Figure 4B) as previously observed (26). From this, we conclude that IPNV immunization does not modify the long-term VH5/JH5 public memory response induced by VHSV immunization.

For VH4/Cτ, there was a lower amplitude in group E2 (VHSV/IPNV), compared to E1 (VHSV only, Figure 4C). This was also shown for the total cumulated expression of Top50 clonotypes in E2 (Figure S3Ak, red bars) which was lower compared to E1 (Figure S3Ae). This was also suggested by the clonotype size distribution (Figure 4C, E1 and E2). A detailed analysis of the VH4/Cτ showed that three fish showed a strong persistent response while one expressed a modest response among the VHSV immunized fish (E1, Figure S4F). In group E2, one fish did not respond at all, one showed a weak response, and two expressed expanded clonotypes at the same level as in group E1 (Figure S4G). While the number of fish is too small to draw firm conclusions, these results may suggest a trend toward a reduction in number of fish showing a persistent VH4/Cτ response typical for VHSV after subsequent IPNV immunization.

### Impact of IPNV Immunization on the Secondary Response to VHSV in VHSV Primed Fish

We were also interested in understanding to what extent IPNV immunization may impact on the secondary (recall) response to VHSV, shown in the groups E3 and E4. Fish were primed and boosted with VHSV (E4), and in parallel, intermittently immunized with IPNV (2x) before being boosted with VHSV (E3).

The analysis of the public VH5/Cμ in group E3 did not reveal any effect of the heterologous viral immunization on the secondary response to VHSV (Figure 4D and Figures S3A,B, panels c vs. i, green bars).

For VH4/Cτ, the secondary response to VHSV in E3 presented as total cumulated expression of Top50 clonotypes (Figure S3Al) was of the same order of magnitude as in group E4 (Figure S3Af), showing that intermittent immunization with IPNV did not alter the total frequency of Top50 clonotypes. The response was partly based on clonotypes shared by several individuals, especially VH4JH1 CARGIQLQFGLFIHAFDYW that was shared by two fish, or VH4JH1 CARGYTVTVWAFYAFDYW shared by 4 fish in group E3, the latter complying with criteria for public clonotypes. The two clonotypes were also highly expressed in two fish in group E2. Clonotype VH4JH1 CARGYTVTVWAFYAFDYW was also highly expressed in two fish in E1, and one fish in E4. Thus, this clonotype was not expanded in all individuals responding to VHSV but was clearly recurrent and specifically involved in the response. We conclude that the secondary VH4/Cτ response elicited by VHSV immunization was as strong in fish intermittently immunized with IPNV, and possibly more convergent (i.e., with a higher expression of shared clonotypes) than observed in groups primed and boosted with VHSV only.

Overall, these observations suggest that secondary IPNV immunization on top of a VHSV priming does not affect the composition and strength of the long-term VH5Cμ public secondary response induced by VHSV. Nevertheless, our data may point to subtle differences in the private VH4/Cτ response after secondary immunization with IPNV but further studies with larger cohorts will be necessary to get definitive documentation.

### IPNV Responses in VHSV Primed Fish

Finally, we looked for signatures of the anti-IPNV response in fish primed with VHSV (E2 and E3). To identify IPNV signatures we focused on VH5/Cμ and VH5/Cτ combinations, which encode public and private anti-IPNV clonotypes, respectively.

Concerning VH5Cμ, we already noticed that the global pattern of expression and sharing was not altered by IPNV immunization post primary VHSV immunization. Searching for IPNV-specific VH5/Cμ public clonotypes, we found that the VH5JH5 sequence CARYTGYAFDYW was highly expressed in all fish from group E3 (FC E3/E0 = 6.4). It was also detected and well-expressed in all fish from group E2 (FC E2/E0 = 4.38). In both groups E2 and E3, this clonotype was expressed at levels comparable to what we observed in fish immunized with IPNV only (group E7). In addition to being one of the main players in the response to IPNV, our findings indicate that it was also selected after IPNV immunization in fish with a pre-existing response against VHSV.

**Figure 5:**
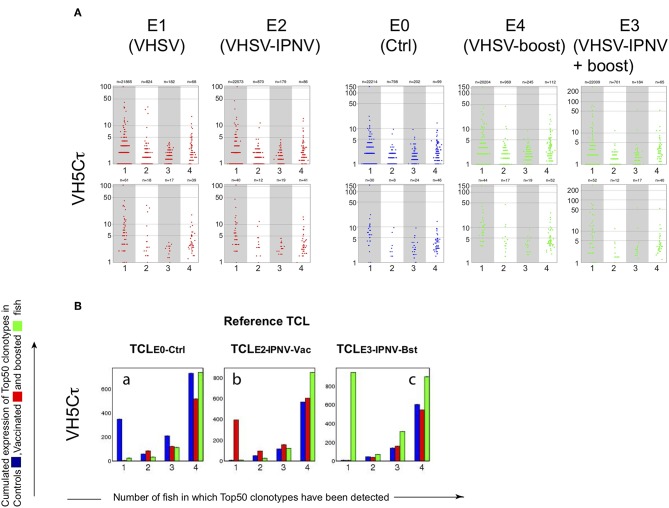
VH5/Cτ repertoires in VHSV vaccinated fish. **(A)** Size distribution of VH5/Cτ clonotypes shared by individual sequenced repertoires from groups E0, E1, E2, E3, and E4. Size distribution is shown for clonotypes found in n control fish (*n* = 1, 2, 3, 4), within a subsampling of 7000 MID per fish. For clonotypes found in several fish, the average size is shown. **(B)** Cumulative expression of Top50 clonotypes shared by n individuals within each group (E0, E2, and E3). For each TCL (TCL-Ctrl, TCL-IPNV-Vac, TCL-IPNV-Bst), bar plots are showing the cumulative expression of TCL clonotypes found in individual subsample(s) from n fish (*n* = 1, 2, 3, 4). Bars are computed from the average values corresponding to top clonotypes found in 1 to 4 fish, over 10 subsamplings of 7000.

A strong reduction of sharing between fish in the VH5/Cτ response, and a significant increase of cumulated expression for Top50 clonotypes (Figures 3I,J, red bars) were observed in fish immunized with IPNV only (E7). We did not observe this response in fish immunized with both VHSV and IPNV (E2 and E3, Figure 5). Distribution of clonotype size (Figure 5A) and cumulated expression were similar for E2 to those of the control group (E0) (Figure 5Bb). The same pattern was observed after boost (Figure 5A, right panels, and Figure 5B; E3).

Thus, we identified the public VH5/Cμ response against IPNV in groups E2 and E3, validating our model for investigating the effect of heterologous immunization on the response induced by VHSV vaccination. This response to IPNV in groups E2 and E3 was comparable to the one observed in group E7 (IPNV immunization only). However, the VH5/Cτ response was lacking, possibly reflecting some interference between the responses to VHSV and IPNV.

## Discussion

Anamnestic responses are the hallmark of adaptive immunity. In both human and mouse, long-term protection is based on the availability of memory cells which can be reactivated by a second encounter with its antigen, and on the persistence of long-lived plasma cells. A growing body of evidence suggest that subsequent infection with heterologous pathogens after vaccination can lead to reduction or “attrition” of established immunological memory and protection. Such mechanisms allowing cells specific for new pathogens to enter the memory compartment, and replace those directed against pathogens previously encountered was first documented for T cells (3, 5). However, there are models showing similar mechanisms affecting the B cell compartments (14–16).

Fish are interesting models to study the effect of heterologous immunization on B cell immunity because Ag specific immune responses observed in these species display a number of important differences compared to mammals. Such differences include a much slower kinetics of Ab responses, especially in cold-water species like rainbow trout. A major increase of serum Ab titers was observed very late after immunization with TNP-KLH+CFA (30 weeks post immunization) in this species (31). The anatomy of lymphoid organs and the micro-environments supporting the persistence of memory cells and long-lived plasma cells are also specific to these organisms (34). In fish, the head kidney is usually the niche for long lived plasma and memory cells, and the spleen is a major site of active responses (17, 31). Finally, receptor diversification (by hypermutation) and clonal selection during B cell responses appear to be less efficient compared to mammals, likely because micro-environments do not allow the fast and powerful selection of cells expressing high affinity Abs (21, 22). Hence, the mechanisms and modalities of heterologous immunity and memory attrition are likely very different in fish and mammals, allowing insightful comparative analyses. Additionally, loss of pre-existing immunity by vaccinated farmed fish is an important issue, in the context of a growing aquaculture increasingly relying on vaccination to limit antibiotics usage and to fight epidemics in farms. For the time being, almost no data exist for heterologous B cell immunity or attrition in fish, especially at the molecular level.

To elucidate the effects of heterologous immunizations in fish, we set up immunization protocols using two natural pathogens of rainbow trout, the rhabdovirus VHSV and the birnavirus IPNV. The strains, doses, and developmental stages used in our experiments guaranteed both efficient Ab responses without adjuvant, and fish survival. IPNV is a relevant candidate for the heterologous pathogen because it is immunologically distinct from VHSV, there is no cross protection by neutralizing Abs or notable cross reactivity. And because it is a ubiquitous key fish pathogen in farming regions. We therefore aimed at testing the consequences of successive heterologous immunizations in a context relatively similar to what may happen in aquaculture.

Altogether our data show that repeated IPNV secondary immunization does not drastically affect pre-existing immunity against VHSV. First, we did not observe any decrease in anti-VHSV antibodies in fish given a secondary IPNV immunization. Since immunity to VHSV largely relies on neutralizing Abs, the sustained anti-VHSV Abs levels indicated that IPNV immunization likely did not affect the protection afforded by vaccination. It remains unclear whether the titer is mainly due to the stability of circulating, neutralizing Abs, or if their production is sustained by ASC. Speculating about stability and half-life of trout IgM is difficult. Stability and half-life is partly determined by the binding strength to their specific epitope, but also by the degree of IgM polymerization (35). Our sequencing approach does not allow the distinction between mRNA encoding secreted and membrane bound IgH. In a previous report, we showed that mRNA for large public clonotypes were almost exclusively encoding secreted IgH chains (34). However, the evolution of the ratio secreted/membrane-bound during the months following immunization could not be directly addressed.

We previously monitored the strong IgM public memory response from VHSV immunization persisting in the spleen several months after vaccination (36). The choice of spleen was based on our previous data reporting a persistent public B cell response detected in this tissue several months post-immunization. While blood likely reflects what we have seen in the spleen, the situation might be different in the kidney which is considered as the niche for plasma cells in fish. Importantly, we did not detect anti-VHSV Ab-producing cells in the kidney 5 months post immunization (before boost). Our preliminary data from the kidney suggest that the kinetics of persistence of cells expressing public clonotypes is rather similar in spleen and kidney. Consistent data were recently reported in Channel catfish immunized with TNP-KLH, in head kidney, spleen, and PBL (37).

Public VH5/JH5 clonotypes involved in this response are expanded in all immune individuals, and it was therefore possible to assess their frequency and to test the effect of a heterologous immunization by IPNV. The systematic expansion of multiple private VH4 IgT clonotypes that occurs in most VHSV vaccinated fish provided another response to which heterologous immunization may have an impact. Our repertoire analyses identified VH/C combinations responding consistently to IPNV immunization, in fish vaccinated against VHSV or not, thus proving that an independent response occurred against IPNV.

VH5JH5 IgM public clonotype frequency and expression measured in spleen 5 months after VHSV vaccination were not modified by the sequential IPNV immunization. The persistent response we previously described in VHSV-vaccinated fish was at this time point still present and did not show any significant shift in its clonotypic composition, suggesting that the splenic pool of Ab-producing cells should be able to accommodate new clonotypes specific to IPNV without drastic attrition of VHSV-specific cells. We have already reported that the public response to VHSV was not dramatically modified 1 month after a boost (i.e., with the same virus) (26). Also, in this context, we found no evidence of any significant effect of IPNV immunization on the public response to the VHSV boost.

We also examined the IgT private response using VH4. This response was frequently observed in VHSV-immunized fish, but typically consists of unrelated expanded clonotypes in different individuals. With 3/4 high responders in E1, and 1/4 in E2, our data may suggest that the response is somewhat lower in IPNV immunized fish. The number of individuals studied, and the private nature of the response, preclude general conclusions. Moreover, there was no visible difference between E3 and E4 groups after the boost. These observations deserve to be extended to larger fish cohorts, but our data show that IPNV does not drastically dampen this response either.

Besides, VHSV-specific ASC (VHSV-ASC) were quantified from head kidney as described earlier (17, 26). They were >20 times more frequent after the boost, both in fish that had been immunized or not with IPNV. VHSV-ASC may appear to be slightly more frequent in E1 (respectively E4) compared to E2 (respectively E3). However, such differences are very small in ELISPOT assays and would need a large number of fish to be properly assessed.

Besides the consequences of heterologous immunization, our ELISPOT data confirms our previous observations of the effect in spleen and kidney afforded by boost (26). In the head kidney, the contrast between low numbers of Ab-producing cells at 150 days post immunization (E1 and E2) and higher numbers in E3 and E4 30 days after boost, shows that boost led to a raise in the number of Ab-producing cells. While anti-VHSV neutralizing Ab titres were not changed significantly by the boost, this difference is somewhat surprising. A compensation between increased production and higher degradation due to the boost might be evoked, but further work with more time points will be needed to clarify this issue.

We also looked further into whether boost, homologous and heterologous, may have modified the composition and the clonotypic diversity of the public response against VHSV in the spleen. We compared different immunization protocols, i.e., combinations of homologous or heterologous immunogens and different timings: (1) fish immunized only once, analyzed 5 months post-immunization with or without additional IPNV immunization [(26) and this work]; (2) fish immunized twice at 3 weeks interval analyzed 3 weeks after boost (34), and (3) fish immunized twice at 5 months interval, analyzed 1 month after boost, with or without additional IPNV immunization [(26) and this work]. Under these conditions, we did not see any clear difference in diversity induced by the second immunization. VH5JH5 public responses after boost did not appear to be “narrower” than after a first immunization. The cumulative expression of public clonotypes increases after boost, as illustrated in Figure S5, but we do not see that one or two public CDR3 are strongly selected after boost or are more frequently expressed. This observation would suggest that there is no predilection for public CDR3 sequences for the virus used for immunization.

Altogether, our data suggest that repeated immunizations with IPNV do not lead to a significant attrition of pre-existing responding B cells specific for VHSV. This observation supports the idea that secondary immunizations with a ubiquitous virus, such as IPNV, would not abolish protective humoral responses elicited by previous vaccinations. However, we have studied one virus only, and further work with other pathogens is needed to clarify whether successive heterologous immunizations could reduce protection induced by primary vaccination, and thus confirm the generality of our findings.

Finally, the response to VHSV monitored in this work is likely based on the persistence of long-lived splenic Ab-producing cells. That said, the nature of “memory cells” in fish remains elusive and understanding mechanisms of heterologous immunity and attrition in these species will require a better definition of their memory lymphocytes.

## Data Availability Statement

The datasets generated for this study can be found in the BioProject NCBI database: SRP128087 and PRJNA530387.

## Author Contributions

SN, SM, PB, and ØE conceived the project. SN, SM, LJ, EQ, NO, HM, PB, and ØE designed experiments. SN, SM, and PB performed wet-lab experiments. SN, SM, LJ, NO, HM, PB, and ØE performed primary data analysis. EQ provided resources. SN, SM, LJ, PB, and ØE wrote the manuscript. All authors edited the manuscript.

### Conflict of Interest

The authors declare that the research was conducted in the absence of any commercial or financial relationships that could be construed as a potential conflict of interest.
