# Supplementary figures and images for: Sequential Immunization With Heterologous Viruses Does Not Result in Attrition of the B Cell Memory in Rainbow Trout
Source: Front Immunol. 2019 Nov 19;10:2687. doi: 10.3389/fimmu.2019.02687 (PMC6882293; doi:10.3389/fimmu.2019.02687)

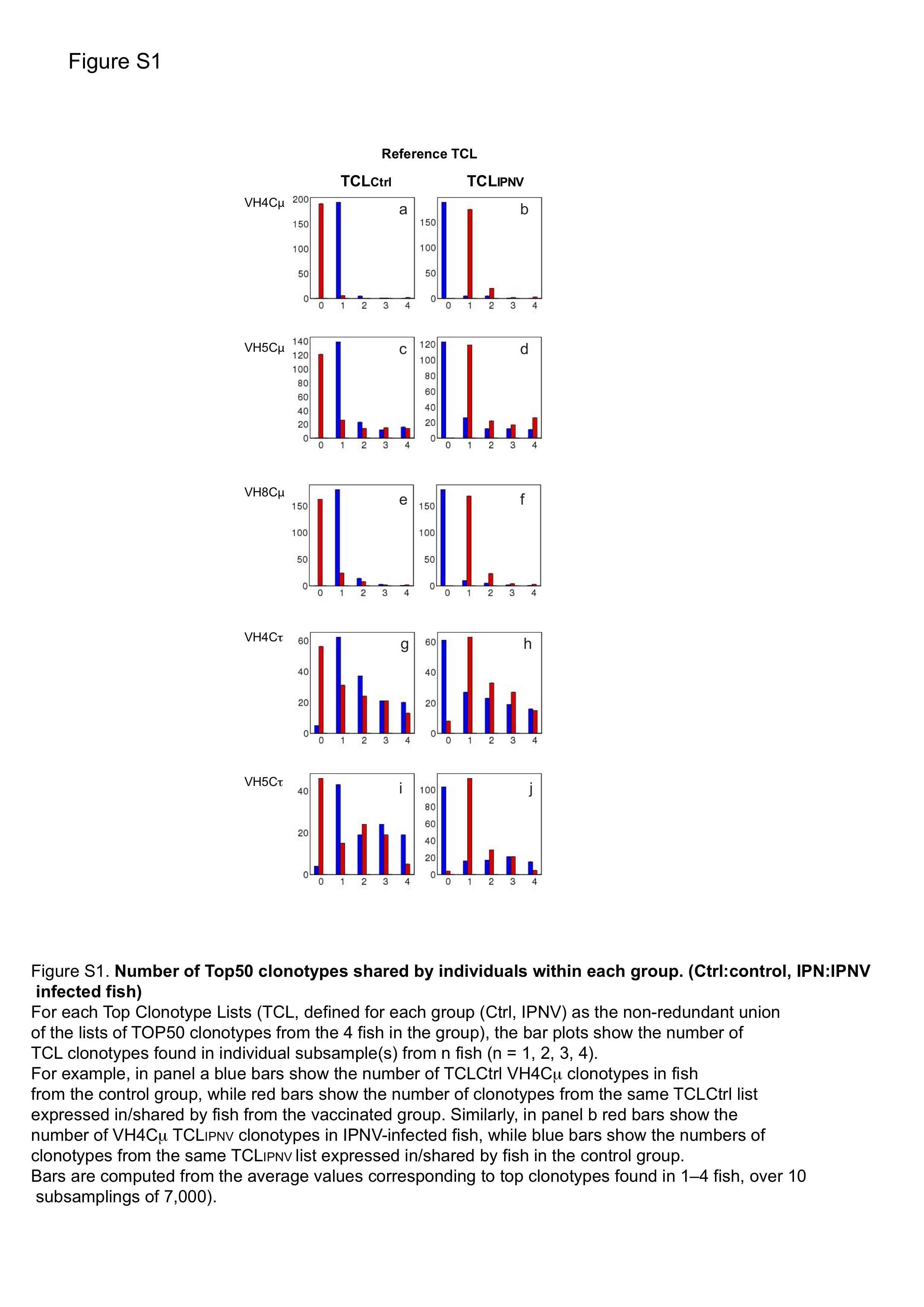

Supplement: Supplementary file 2 [file Image_1.TIFF]

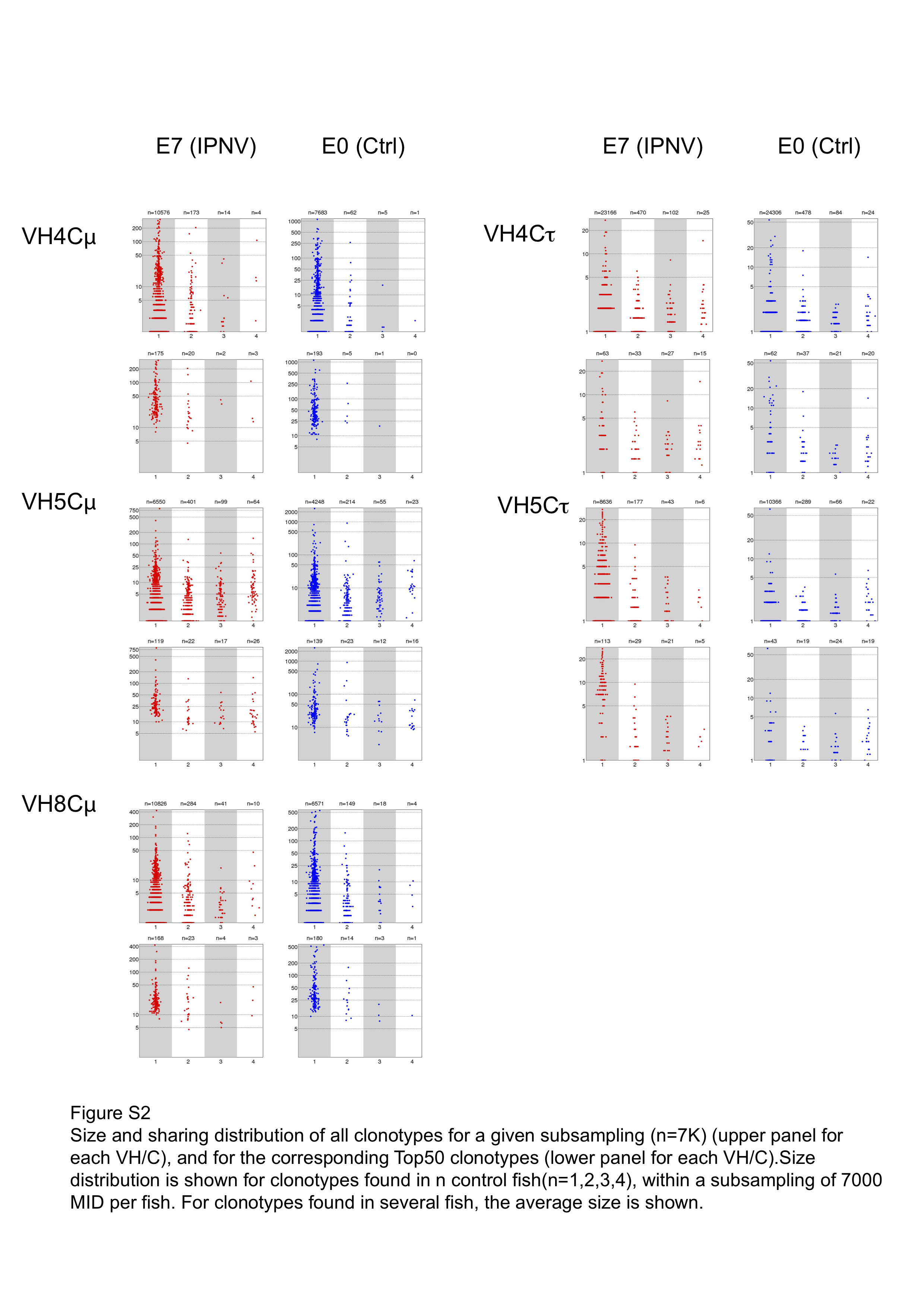

Supplement: Supplementary file 3 [file Image_2.TIFF]

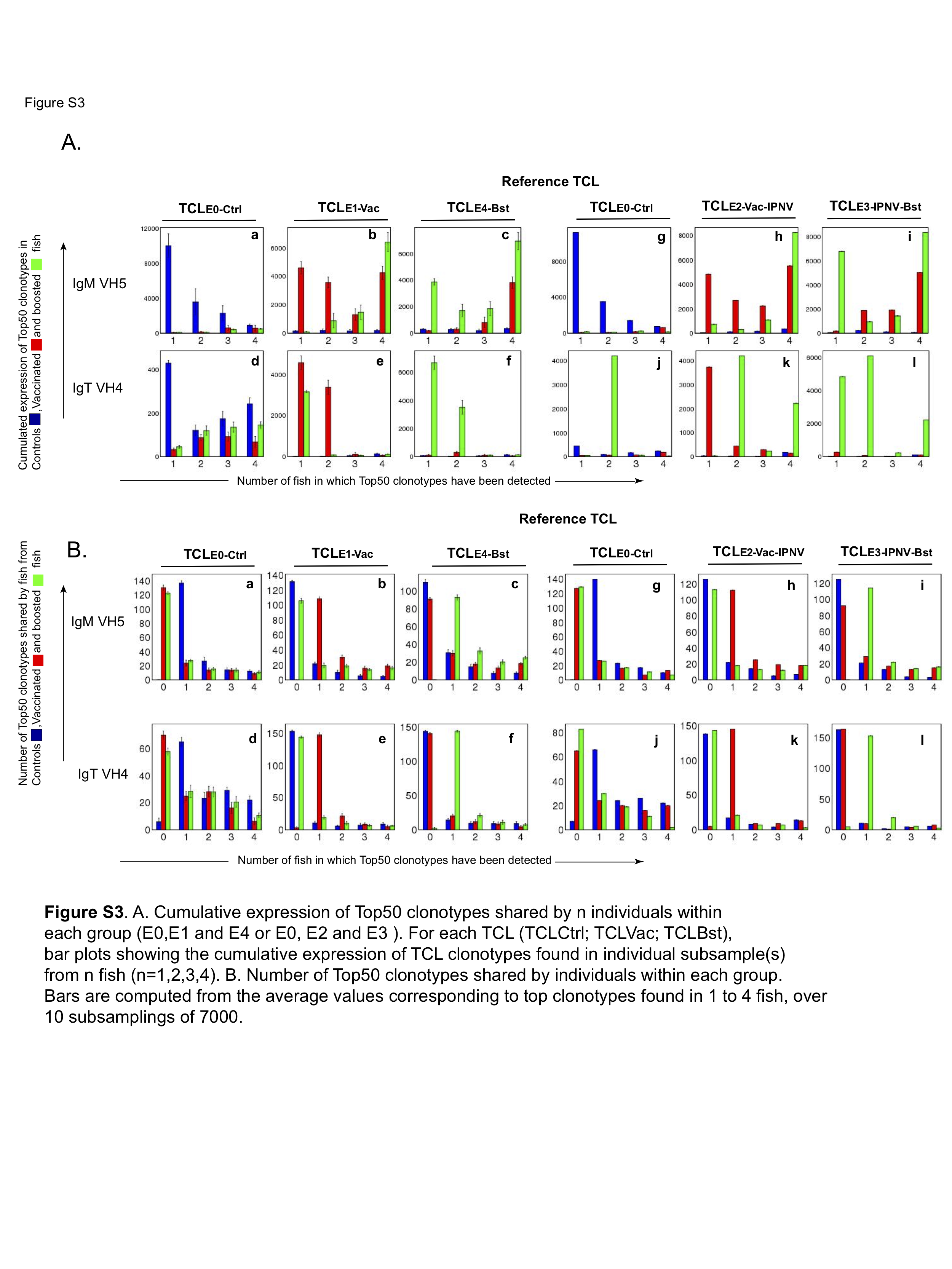

Supplement: Supplementary file 4 [file Image_3.TIFF]

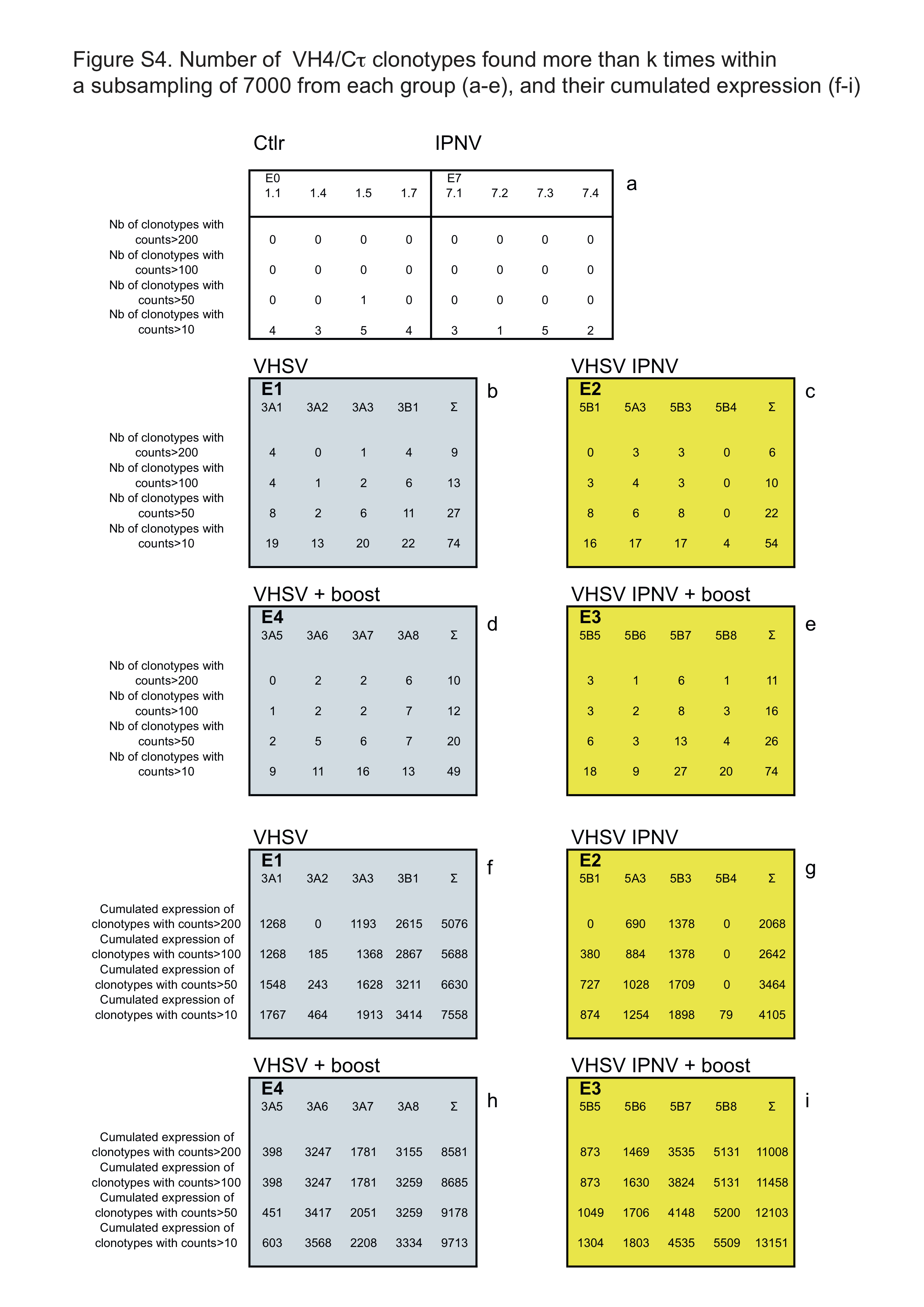

Supplement: Supplementary file 5 [file Image_4.TIFF]

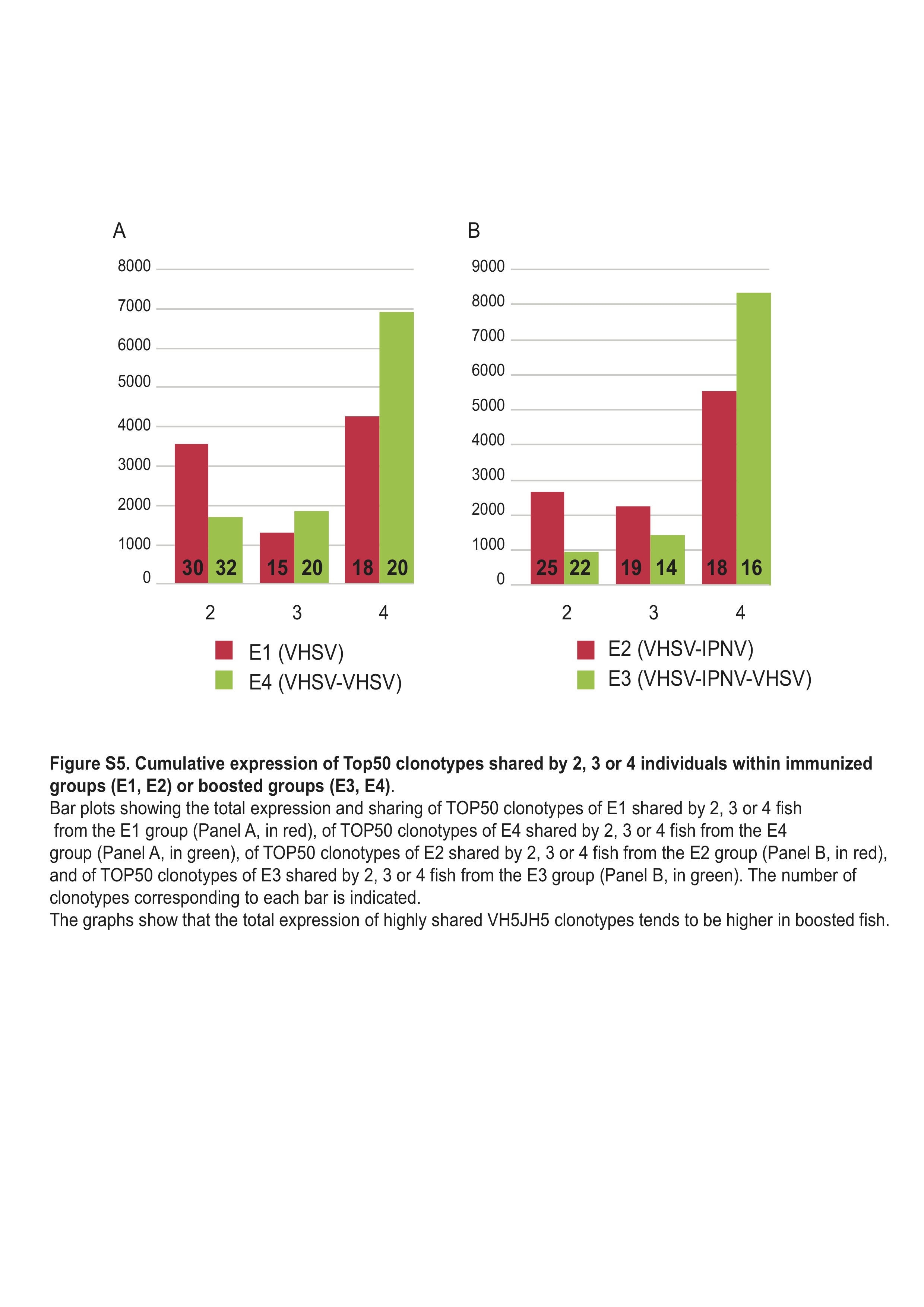

Supplement: Supplementary file 6 [file Image_5.TIFF]
